# Effect of Pulsed Electric Field Pretreatment on the Texture and Flavor of Air-Dried Duck Meat

**DOI:** 10.3390/foods14111891

**Published:** 2025-05-26

**Authors:** Ning Zhang, Zihang Shi, Yangyang Hu, Yangying Sun, Changyu Zhou, Qiang Xia, Jun He, Hongbing Yan, Hui Yu, Daodong Pan

**Affiliations:** 1 College of Food Science and Engineering, Ningbo University, Ningbo 315211, China; 2 Hangzhou Dakang Pickled Food Co., Ltd., Hangzhou 311107, China; 3 Zhejiang Shanli Foods Co., Ltd., Lishui 323000, China

**Keywords:** pulsed electric field, air-dried duck, texture profile, flavor profile, protein hydrolysis

## Abstract

Pulsed electric field (PEF), a novel non-thermal processing technology, shows great potential in meat processing by regulating macromolecule metabolism and food quality. This study examined the effects of PEF pretreatment at varying electric field strengths (1, 2, and 3 kV/cm) and durations (30, 60, and 90 s) on the color, texture, moisture distribution, free amino acids, and flavor compounds in air-dried duck meat. PEF pretreatment significantly increased brightness (*p* < 0.05), while PEF treatment (3 kV/cm, 30 s) improved the textural properties of air-dried duck meat, reducing chewiness and hardness by 65.44% and 59.97%, respectively. It promoted myofibril disruption and vacuolization, reducing water mobility and improving moisture retention. Enhanced endogenous enzyme activity under PEF facilitated protein degradation, boosting total free amino acid content, particularly umami and sweet amino acids (glutamic acid, alanine). PEF pretreatment also elevated key aroma compounds, such as hexanal, methyl caprate, and 4-methyl valerate, improving the flavor profile of air-dried duck meat. This study provides technical support for integrating PEF technology into traditional poultry processing.

## 1. Introduction

Air-dried duck meat is one of the classic Chinese cured and preserved goods, which is created by cleaning, curing, air-drying, and packing fresh or thawed duck [1]. Air-dried duck meat is popular among consumers because its endogenous enzymes decompose proteins and fats to produce a variety of flavor substances to give the product a unique and attractive flavor [2]. Traditional processing methods have clear drawbacks even if they ensure the development of the distinctive flavor of air-dried duck meat. Firstly, the production capacity is limited, and the complete processing cycle of air-dried duck meat is up to several weeks, with high artificial dependence and low production efficiency [1]; secondly, it is challenging to guarantee the consistency of the quality of the air-dried duck meat because the various kinds, sizes, and air-drying conditions of the raw materials cause variations in the quality of the air-dried duck meat; at the same time, due to the long air-drying time, nutrients such as soluble proteins and vitamins in the muscle tissues are prone to irreversible losses. Therefore, it is of great scientific value and practical significance to explore new technologies that can not only ensure that the distinctive flavor of air-dried duck meat is formed but also enhance processing stability to support the enhancement of air-dried duck meat quality.

In the coming years, a non-thermal food processing technique called pulsed electric field (PEF) technology will use parallel electrodes to send brief, high-voltage pulses to the food substrate, improving energy efficiency and product quality [3]. Research has demonstrated that this technique improves food color, flavor, and nutrients [4]. PEF technology was first used mostly in the non-thermal sterilization process of liquid food products, such as dairy products [5], fruit juice products [6], and other fields. With further research, its application has been expanded to solid foods, including meat [7], fruits and vegetables [8], and aquatic products [9]. In the field of meat processing, PEF technology is mainly applied to improve tenderness [10] and assist marination [11], digestibility [12], and thawing [13]. To the best of our knowledge, there is no research on how PEF treatment affects the formation of flavor in meat products. Additionally, the majority of PEF technology studies that are currently available concentrate on the impact of a single parameter (such as the number of pulses or the strength of the electric field), and there are not many systematic studies on the combined effects of several factors.

Therefore, in this study, PEF technology was employed in the preparation of air-dried duck meat to see how it affected the texture and flavor. The effects of different electric field intensity (1, 2, and 3 kV/cm) and treatment time (30, 60, and 90 s) combinations on the texture and water retention of air-dried duck meat were first investigated. Using low-field nuclear magnetic resonance, the impact of PEF treatment on the moisture distribution in air-dried duck meat was examined. The impact of PEF treatment on endogenous enzyme activities, protein hydrolysis, and free amino acid profiles of air-dried duck meat was determined. GC-MS was used to examine how the PEF treatment affected the flavor of the air-dried duck meat. This study offers theoretical direction for producing air-dried duck meat products of superior quality.

## 2. Materials and Methods

### 2.1. Materials

Pekin ducks (average rearing cycle of 60 d; average weight of about 2.5 kg) were purchased from Ningbo Lande Company (Ningbo, Zhejiang, China). A high-voltage pulsed electric field sterilization device (DTPEF-1501, Dalian Dingtong Science and Technology Development Co., Ltd., Dalian, Liaoning, China) was used to treat the duck meat. Food-grade sodium chloride was purchased from Zhejiang Tianhe Food Biological Company (Tongxiang, Zhejiang, China). 2-methyl-3-heptanone (99% pure, used as internal standard), Triton X-100 (98% purity), and Brij-35 (99% purity); Z-Arg-Arg-AMC (95% purity) and Z-Phe-Arg-AMC (95% purity); and Suc- LLVY-AMC (95% purity) were purchased from Merck (Darmstadt, Germany), McLean (Shanghai, China), and Yuan Ye (Shanghai, China), respectively. The remaining substances utilized in this investigation were at least analytical grade.

### 2.2. Sample Preparation

After being cleansed and any surface blood or water dried off, the fresh Peking ducks were put in the pulsed electric field (PEF) device’s treatment chamber. The electric field intensity, pulse frequency, and duty cycle were set to 1, 2, and 3 kV/cm, 125 Hz, and 50%, respectively, and the pulse treatment time was set at 30, 60, and 90 s, and the number of pulses was set at 1875, 3750, and 5625. Fresh Peking ducks not pretreated by PEF were selected to serve as the control group. Following PEF pretreatment, the ducks in each treatment group (untreated, CK; 1 kV/cm, 30 s, LPEF-30; 1 kV/cm, 60 s, LPEF-60; 1 kV/cm, 90 s, LPEF-90; 2 kV/cm, 30 s, MPEF-30; 2 kV/cm, 60 s, MPEF-60; 2 kV/cm, 90 s, MPEF-90; 3 kV/cm, 30 s, HPEF-30; 3 kV/cm, 60 s, HPEF-60; and 3 kV/cm, 90 s, HPEF-90) were submerged in an 8% (*w*/*v*) brine solution, marinated for 24 h at 4 °C, and then allowed to air-dry in an air-drying box for 4 d at 16 °C and 68% humidity. At the conclusion of the air-drying procedure, the breast meat of the ducks was collected, sealed, and kept at −80 °C in a refrigerator for further experimental analyses.

### 2.3. Determination of Color

A calibrated colorimeter (SWG-2300, Shanghai Shuo Guang Electronic Technology Co., Ltd., Shanghai, China) was used to measure the color of air-dried duck meat samples, which quantifies three key color parameters: L* (brightness), a* (redness), and b* (yellowness). Instrument calibration is performed prior to measurement: First, perform zero calibration by pressing the measuring port of the instrument firmly onto the matching black cylinder, then press and hold the “Cal” key until “Zero Cal Completed” is displayed. Then, use the original standard white plate to calibrate, press the measuring port flat to ensure there is no light leakage, and then calibrate again. To ensure representative sampling, five different surface locations were selected for color determination for each meat sample.

### 2.4. Determination of Textural Profile

For texture profile analysis, a texture analyzer (TA. XT Plus, Stable Micro Systems, Surrey, UK) fitted with a P50 cylindrical probe was used to measure the 2 × 2 × 2 cm cubes of air-dried duck meat samples. The instrument parameters were set as follows: pre-test speed of 2 mm/s, mid-test speed of 1 mm/s, post-test speed of 2 mm/s, compressive strain of 30%, and compression interval of 5 s [14]. To assure data reliability, every sample underwent three independent tests, and the three key indexes of hardness, gumminess, and chewiness were selected for analysis.

### 2.5. Determination of Moisture Content

To determine moisture content, 1.0 g of air-dried duck meat was accurately weighed and analyzed using a fully automated moisture meter (FM-971, Dongguan Fan Ma Electronic Technology Co., Ltd., Dongguan, China). Three measurements were performed to ensure the reliability of the data.

### 2.6. Low-Field Nuclear Magnetic Resonance (LF-NMR)

The approach provided by [15] was slightly modified. The moisture distribution in air-dried duck meat samples from various treatment groups was measured using an LF-NMR analyzer (NMI20-060H-1, Neumay, Suzhou, China). The proton resonance frequency was 23.4 MHz, and the measurement temperature was set at 32 °C. In each investigation, 2.5 g of the sample was loaded into an NMR tube with a 25 nm diameter. With the following parameters, a Carr–Purcell–Meiboom–Gill (CPMG) pulse sequence was used to calculate the transverse relaxation time (T2): 200 kHz spectrum width, 0.002 ms RF delay time, 6000 ms wait time, 0.2 ms echo time, and 12,000 echoes. Multi-Exp Inv analysis software (Niumag Electric Corp, Suzhou, China) was used to process the collected relaxation data.

The proton density distribution in air-dried duck meat was computationally simulated by a magnetic resonance imaging (MRI) system. The imaging parameters were precisely set as follows: the repetition time (TR) was 2800 ms; the echo time (TE) was 33.71 ms [16].

### 2.7. Scanning Electron Microscopy (SEM)

The microstructure of air-dried duck meat samples was examined with a scanning electron microscope (S-3400 N, Hitachi, Tokyo, Japan), following the approach described by [17]. The samples were cut into 0.5 × 0.5 × 0.5 cm cubes, and then the air-dried duck meat was cured for 48 h in a 2.5% (*v*/*v*) glutaraldehyde solution. Subsequently, a gradient of 30%, 50%, 70%, 80%, 90%, and 100% (*v*/*v*) ethanol solution was used to elute the air-dried duck meat. The samples were then freeze-dried in a vacuum and plated with a gold treatment. Finally, the air-dried duck meat tissue structure was observed at a magnification of 1000×.

### 2.8. Protease Extraction and Activity Determination

#### 2.8.1. Extraction of Crude Enzyme Solutions

With minor adjustments, the method of [18] was used to carry out the crude enzyme extraction of cathepsin-B and cathepsin-B+L. An amount of 0.5 g of air-dried duck meat was weighed, and 2.5 mL of 50 mM sodium citrate buffer (pH 5.0, 0.2% Triton X-100, 1 mM EDTA) was used to homogenize the sample at 10,000 rpm for 30 s. The material was centrifuged at 12,000× *g* for 20 min at 4 °C. The supernatant was then filtered through a glass sponge to determine enzyme activity. The extraction methods were carried out on ice to ensure the enzyme’s stability.

Calpain crude enzyme extraction was performed in accordance with the method described by [19]. To determine calpain activity, 3.0 mL of 50 mM Tris buffer (pH 8.5, 3 mM EDTA, and 10 mM β-mercaptoethanol) was mixed with 1.0 g of air-dried duck meat that had been weighed. The mixture was centrifuged at 12,000× *g* for 20 min at 4 °C after being homogenized in an ice bath (10,000 rpm, 30 s). Glass wool was used to filter the supernatant. To ensure the enzyme’s stability, the extraction steps were carried out on ice.

#### 2.8.2. Determination of Cathepsin-B, Cathepsin-B+L, and Calpain Activities

Z-Arg-Arg-AMC and Z-Phe-Arg-AMC were used as substrates to assess the activities of cathepsin-B and cathepsin-B+L, respectively. An amount of 50 μL of enzyme extract was incubated with 250 μL of reaction buffer (pH 6.0, 50 mM phosphate buffer, 0.4 mM EDTA, 0.1% Brij-35, 2 mM DTT, and 0.05 mM specific substrate) at 37 °C for 30 min. Fluorescence was continuously monitored using a multifunctional enzyme marker (Tecan Infinite 200 PRO, Tecan Group Ltd., Männedorf, Switzerland) with 380 nm and 440 nm as the excitation and emission wavelengths.

For calpain, Suc- LLVY- AMC was used as the substrate for the activity assay. An amount of 50 μL of crude enzyme extract was mixed with 250 μL of reaction buffer (pH 7.5, 100 mM Tris-HCl, 1 mM DTT, and 0.05 mM of specific substrate). Next, the mixture was incubated at 37 °C. Using a multifunctional enzyme marker (Tecan Infinite 200 PRO, Tecan Group Ltd., Männedorf, Switzerland) and excitation and emission wavelengths of 355 and 460 nm, respectively, fluorescence was continually observed.

### 2.9. Determination of Volatile Flavor Compounds

Headspace solid-phase microextraction (HS-SPME) and gas chromatography–mass spectrometry (GC-MS) (Agilent, Santa Clara, CA, USA) were used to detect flavor substances in air-dried duck meat. After precisely weighing 5.0 g of air-dried duck meat, it was placed in a 20.0 mL headspace extraction vial together with 10.0 μL of internal standard solution (2-methyl-3-heptanone at 10 ppm). After inserting an SPME extraction head (Supelco, Inc., Bellefonte, PA, USA), headspace extraction was performed for one hour at 50 °C. Following extraction, the SPME extraction head was placed into the GC system’s injection port and resolved for 4 min at 250 °C. A VOCOL capillary column (60 m × 0.32 mm × 1.8 μm) (Agilent, CA, USA) was chosen, and the following parameters were established for detection: high-purity helium was employed as the carrier gas, and its flow rate was set at 1.0 mL/min; the column chamber was first heated to 40 °C and kept there for 5 min, then it was heated to 200 °C at 5 °C/min; and finally, it was heated to 250 °C at 15 °C/min and kept there for 8 min. The scanning mass range was set at 35–350 u, the electron energy was set at 70 eV, and the ion source’s temperature was set at 230 °C. Volatile compounds were qualitatively analyzed by comparison to the NIST 14 database (NIST, Gaithersburg, MD, USA) analysis. Volatile compounds were then quantitatively analyzed by dividing the area of each compound by the area of the internal standard [20].

### 2.10. Determination of Free Amino Acids (FAAs) and Taste Activity Value (TAV)

The method of Roobab et al. (2023) [21] was referenced in this study. Briefly, 10.0 g of the sample was weighed, mixed with 10.0 mL of ultrapure water, homogenized, and processed. The supernatant was collected, and 1.0 mL was combined with 1.0 mL of 8% sulfosalicylic acid solution. After mixing, the mixture was centrifuged at 10,000 rpm for 15 min. The supernatant was removed, blow-dried, and then resolubilized by adding HCl. The resulting solution was filtered, and free amino acids (FAAs) were quantified using an automatic amino acid analyzer (Hitachi L-8900, Hitachi Co., Ltd., Tokyo, Japan).

TAV describes the taste intensity of a food and is the ratio of the amount of a taste-presenting substance in the food to its taste threshold. When TAV > 1, the flavor-presenting substance contributes to the flavor presentation of the food, and the larger the value, the greater the contribution. The formula is as follows:

TAV = C/T

C: concentration of the flavor-presenting substance; T: taste threshold of the flavor-presenting substance.

### 2.11. Statistical Analysis

Every experiment was conducted independently, and data analysis required at least three replications. The mean ± standard deviation was used to present the results. In SPSS 22.0 (IBM SPSS Statistics 22, IBM Co., Chicago, IL, USA), the data were assessed using one-way analysis of variance (ANOVA) and Duncan’s multiple comparisons, with significant differences defined at *p* < 0.05. Origin 8.0 (Origin Lab Corp., Northampton, MA, USA) was used to make the graphs.

## 3. Results and Discussion

### 3.1. Color Analysis

Meat color is a key quality indicator that influences customer purchase decisions and food product acceptability [22]. As shown in Table 1, the color analysis results showed that the L* parameter of the air-dried duck meat was significantly affected (*p* < 0.05) by PEF treatment. L* increased by 6.77 in the LPEF-30 group compared to the CK group, while increases by 0.56 and 2.86 in the LPEF-90 and HPEF-30 treatments were noticeably less than those in the CK group. These results imply that the electric field intensity and PEF treatment duration had an inverse connection with the surface brightness of air-dried duck meat; the L* values tended to decrease with increasing electric field intensity and treatment duration. The a* values in the PEF-treated group were higher than those in the CK group, which could be due to enhanced light scattering by electroporation, highlighting the red color. However, the b* values were not affected by the PEF treatment. The observed color changes could be explained by the effects of the PEF treatment on the moisture distribution and content of the air-dried duck meat surface, which in turn affected the amount of light reflected and absorbed off the product surface [23].

### 3.2. Texture Profile Analysis

Texture profiles are a significant quality indication during meat processing that can influence the tissue state and sensory quality of meat products [24]. As shown in Table 2, the texture profile results indicated that PEF treatment significantly affected the hardness, chewiness, and gumminess of air-dried duck meat (*p* < 0.05). According to the analysis, the hardness, chewiness, and gumminess of air-dried duck meat decreased as the electric field strength increased. At an electric field strength of 3 kV/cm, the hardness, chewiness, and gumminess could be decreased by as much as 65.44%, 59.97%, and 60.57%, respectively. It is worth noting that higher electric field intensity would destroy the myogenic fiber structure of air-dried duck meat, resulting in too loose a tissue structure and affecting the texture of the products, thus deteriorating the sensory quality of the samples in the HPEF-treated group. Furthermore, a positive correlation was found between PEF treatment duration and textural measures. This was due to the fact that as the processing time increased, it caused the meat product to lose water, making the meat slightly tougher [25]. It was found that the hardness and chewiness of meat products were negatively correlated with tenderness values [12]. PEF treatment accelerated meat tenderization by activating calpain, which in turn improved the textural properties of air-dried duck meat. This corroborates with SEM observations that PEF treatment improves the textural properties of air-dried duck meat by destroying their muscle fiber structure and that the electroporation impact of PEF treatment is more pronounced in destroying the tissue structure with the electric field strength and treatment time rise. Compared with the LPEF group, the MPEF and HPEF groups of air-dried duck meat had less hardness and chewiness and softer texture, but the HPEF group had too loose tissue structure and poor character; therefore, the samples from the MPEF-treated group were selected not only to improve the textural characteristics of air-dried duck meat but also to maintain the structural integrity of the muscle fibers.

### 3.3. Moisture Content Analysis

Textural changes in air-dried duck meat are closely related to moisture content. Table 2 shows the effect of the PEF treatment on the moisture content of air-dried duck meat. According to the analysis, the PEF-treated duck meat had a considerably higher moisture content (*p* < 0.05) than the CK group. The moisture content of duck meat treated with PEF was 5.55–9.50% greater than that of the CK group. The significant increase in moisture content of air-dried duck meat could be attributed to the PEF-induced electroporation effect, which enlarged the interstices of muscle fibers and strengthened the interaction forces between moisture molecules and protein molecules, improving the water-absorbing capacity of the meat. Meanwhile, moisture migration in air-dried duck meat led to changes in the spatial structure of their proteins, which affected the textural properties of muscle tissues. This positively affects the overall texture of air-dried duck meat products.

### 3.4. Moisture Distribution

**Figure 1:**
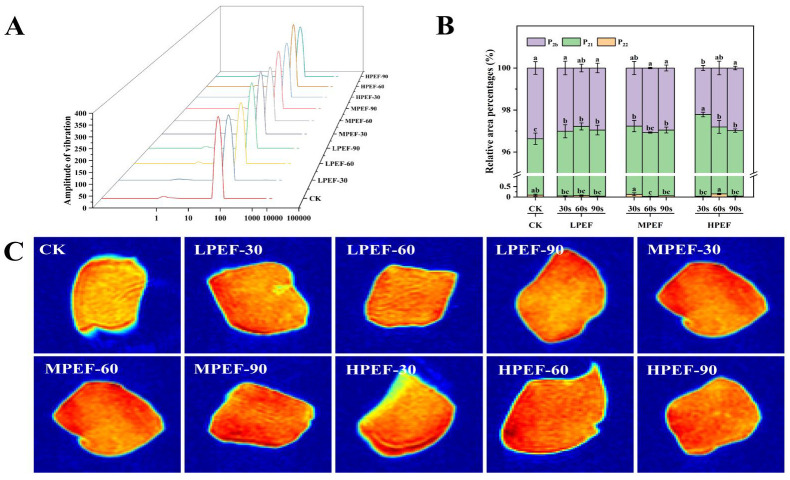
The effect of PEF treatment on the moisture distribution in air-dried duck meat. T2 relaxation spectra (**A**); relative peak area percentages (**B**); and magnetic resonance images (**C**). Data are expressed as mean ± standard deviation. Different letters (a–c) indicate significant differences in one-way ANOVA and Duncan’s multiple comparisons (*p* < 0.05). CK: untreated; LPEF-30: PEF treatment (1 kV/cm, 30 s); LPEF-60: PEF treatment (1 kV/cm, 60 s); LPEF-90: PEF treatment (1 kV/cm, 90 s); MPEF-30: PEF treatment (2 kV/cm, 30 s); MPEF-60: PEF treatment (2 kV/cm, 60 s); MPEF-90: PEF treatment (2 kV/cm, 90 s); HPEF-30: PEF treatment (3 kV/cm, 30 s); HPEF-60: PEF treatment (3 kV/cm, 60 s); and HPEF-90: PEF treatment (3 kV/cm, 90 s).

The water distribution states in air-dried duck meat from different treatment groups were analyzed by LF-NMR measurements. Figure 1 shows three states of water: bound water (T_2b_, 1–10 ms), not easily flowable water (T_21_, 10–100 ms), and free water (T_22_, 100–1000 ms). Among these results, the peak area of T_21_, which represented not easily flowable water, was the largest, which was in line with water accounting for more than 90% of the total muscle water content [26], which is closely related to the water retention capacity of the meat. It was found that the proportion of water not readily available for movement (P_21_) was significantly higher in the PEF-treated group compared with the CK group (*p* < 0.05). This change in water distribution suggests that the fixation of water within the muscle fiber structure was enhanced, reducing water freedom. Thus, PEF treatment contributed to the improvement in the water retention capacity in air-dried duck meat. This is in line with earlier research by [7], which discovered that PEF treatment affected the production of free radicals of the polar groups of proteins and induced the unfolding of protein molecules, which in turn increased the peak area ratio of T_21_ and improved the water retention of the meat. However, P21 showed a decreasing trend with increasing treatment time, which could be attributed to the cumulative energy input of longer PEF treatments leading to protein denaturation and aggregation, and this alteration in protein conformation compromises the water-binding sites in the myofibril network. The proton density and moisture distribution of food matrices can be mapped using magnetic resonance imaging (MRI) [27].

The MRI images of air-dried duck meat from various treatment groups are displayed in Figure 1C. The color shift from blue to red signifies a change in hydrogen proton density; the darker the red, the stronger the hydrogen proton signal and the higher the associated water content. Due to the preferential dehydration of the duck meat surface during the air-drying process, the figure shows that the hydrogen proton signal intensity in the peripheral region of the air-dried duck meat of various treatment groups was significantly lower than that in the internal region. It was observed that the PEF treatment could improve the water retention of air-dried duck meat because the hydrogen proton signals of the PEF-treated samples were noticeably stronger (deepened red color) than those of the CK group. When an electric field strength of 2 kV/cm was applied, the samples in the MPEF-treated group displayed notably higher signal intensity and more uniform signal distribution, indicating that the water content in the air-dried duck meat was higher and the water distribution was more uniform. Combined with the T_2_ chromatograms and MRI observations, it is jointly shown that PEF treatment (especially in the MPEF group) improved the moisture distribution and water retention of the air-dried duck meat by disrupting their textural properties.

### 3.5. SEM Analysis

**Figure 2:**
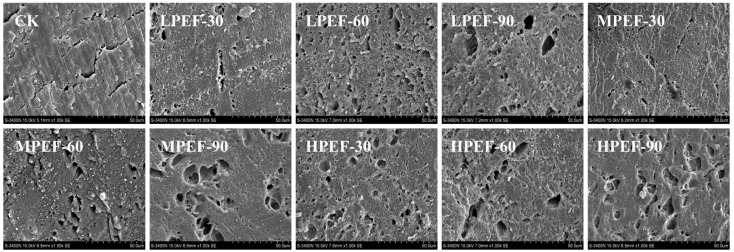
Scanning electron microscope images (magnification 1000×).

The microstructure of air-dried duck meat can be observed by SEM, and the integrity and arrangement of the microstructure of muscle fibers is an important factor in determining the texture of meats 2021 [28]. As presented in Figure 2, the samples in the CK group showed a complete tissue structure, with muscle fibers tightly aligned and no obvious gaps between fibers. In contrast, the PEF-treated samples exhibited larger muscle fiber gaps. The electroporation effect and mechanical effect of PEF, which disturbed the tissue structure and widened the muscle fiber voids and gaps, caused the number of circular pores and voids in the muscle fibers to grow as the electric field intensity and treatment duration increased. Similar results were found by [29], where PEF treatment was able to disrupt the muscle fiber structure of beef and widen the gaps between fiber bundles. In addition, larger electric field intensities (e.g., 3 kV/cm) and longer treatment times (e.g., 90 s) can lead to excessive loosening of the tissue structure of air-dried duck meat, which in turn produces undesirable textural characteristics and affects product quality.

### 3.6. Cathepsin-B, Cathepsin-L, and Calpain Activities Analysis

**Figure 3:**
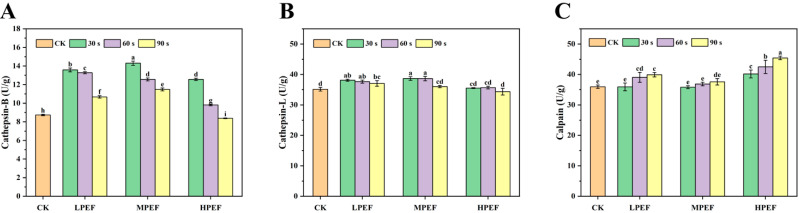
The effect of PEF treatment on the endogenous protease activity of air-dried duck meat. Cathepsin-B (**A**); cathepsin-L (**B**); and calpain (**C**). Data are expressed as mean ± standard deviation. Different letters (a–e) indicate significant differences in one-way ANOVA and Duncan’s multiple comparisons (*p* < 0.05).

Endogenous proteases have been found to play a key role in meat tenderization and muscle protein degradation [19,30]. Cathepsin-B and cathepsin-L are important endogenous proteases in muscle, helping to degrade muscle proteins and produce FAAs and other taste chemicals [31,32] discovered a positive association between total FAA content and cathepsin-B activity in dried grass carp. In comparison to the CK group, PEF treatment markedly elevated cathepsin-B, cathepsin-L, and calpain activities in air-dried duck meat (*p* < 0.05) (Figure 3). This could be attributed to the electro-penetrating effect of PEF treatment, which increased the permeability of cell membranes and promoted the release of endogenous proteases to accelerate protein degradation. Cathepsin-B, cathepsin-L, and calpain showed the highest activities up to 14.30 U/mg, 38.67 U/mg, and 45.43 U/mg, which were 63.71%, 10.04%, and 26.33% greater than that of the CK group. Notably, the activities of cathepsin-B and cathepsin-L decreased with increasing treatment time, which was attributed to the fact that prolonged pulsed electric field treatment disrupts the structure of the protease, leading to a decrease in tissue protease activity. Calpain is essential for the tenderization of meat, according to existing research, and the increase in calpain activity because PEF treatment disrupts muscle fiber integrity, which in turn alters the protein conformation and activates the release of intracellular calpain [33], which is in agreement with the textural and SEM results.

### 3.7. Volatile Flavor Compounds

**Figure 4:**
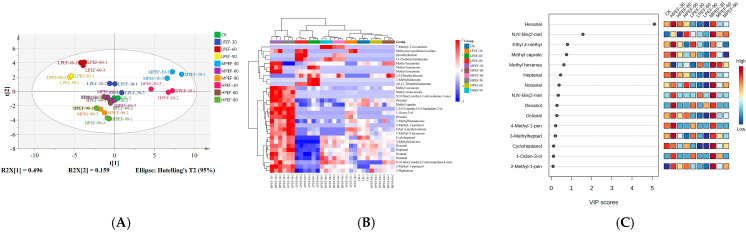
The effect of PEF treatment on volatile flavor substances in air-dried duck meat. GC–MS partial least squares discriminant analysis plot (PLS–DA) (**A**); volatile flavor substances heat map (**B**); and Variable Importance for Projection (VIP) score plot (**C**).

To more thoroughly investigate the impact of PEF treatment on the formation of volatile flavor components in air-dried duck meat, HS-SPME and GC-MS were used to identify and analyze the volatile flavor compounds in samples of air-dried duck meat from various treatment groups. According to Figure 4A, it was found that the samples of each treatment group showed obvious discrete states on the two-dimensional spatial distribution map, indicating that the scent compositions of the air-dried duck meat from various treatments varied. The thermogram in Figure 4B and Table S1 show that 31 flavor compounds, including 4 ketones, 5 aldehydes, 7 esters, 7 alcohols, and 8 hydrocarbons, were detected and identified in the air-dried duck meat. It was discovered that samples from different treatment groups differed considerably in the types and amounts of volatile flavorings (*p* < 0.05). In contrast to the CK group, the flavor complexity of PEF-treated air-dried duck meat was enhanced, especially in the shorter treatment time (30 s); moreover, the contents and types of the characteristic flavor substances (hexanal, methyl hexanoate, and 1-octen-3-ol) of the air-dried duck meat were increased, and the flavor was richer, but the contents of flavor substances (e.g., aldehydes) in the air-dried duck meat were significantly decreased as the treatment time increased. In addition, the content of flavor substances (e.g., esters, alcohols) increased with increasing electric field strength. This is because appropriate PEF treatment enhances the activity of endogenous enzymes and promotes protein degradation, which in turn affects the production of volatile flavor substances in air-dried duck meat; however, PEF treatment destroys the structure of endogenous enzymes and decreases their activity as the strength of the electric field and treatment time increase. Therefore, variations in the amount of alcohols, esters, and aldehydes in air-dried duck meat could be the primary cause of the flavor variations.

The flavor of meat products is greatly enhanced by aldehydes, which are mostly produced by lipid oxidation and have a low threshold and high content [1]. The aldehydes detected in air-dried duck meat were hexanal, heptanal, octanal, and nonanal, with hexanal having the highest content. In the air-dried duck meat of the MPEF-30 and HPEF-30 groups, the contents of hexanal (grass, tallow, and fat flavors), nonanal (fat, citrus, and green flavors), and octanal (fat, soap, lemon, and green) [34,35] were significantly greater than other treatment groups (*p* < 0.05), suggesting that PEF treatment improved the flavor quality of air-dried duck meat by encouraging lipid oxidation and raising the content of aldehydes. The low PEF treatment time (30 s) encouraged the production of aldehydes.

A significant factor in the development of flavor in meat products, the esters created by the esterification reaction between carboxylic acids and alcohols can also produce pleasing flowery and fruity flavors. In contrast to the CK group, PEF treatment enriched the kinds of esters (methyl nonanoate, methyl tri-decanoate) and considerably enhanced the content of esters (methyl caproate, methyl caprylate) in air-dried duck meat (*p* < 0.05).

Alcohols are usually produced by the oxidative breakdown of lipids and the threshold of unsaturated alcohols is usually lower than that of saturated alcohols, which have a greater effect on the flavor of meat products. The main alcohols detected in air-dried duck meat were 1-octen-3-ol, cycloheptanol, and 4-methyl-1-pentanol. The highest alcohol content was detected in the MPEF-30 group, and the unsaturated alcohol 1-octen-3-ol was the most abundant alcohol in air-dried duck meat, which was able to impart mushroom flavor to air-dried meat products and play an important role in the overall flavor of air-dried duck meat. In addition, ketones (2-heptanone) could also give pleasant sweet and fruity flavors to the air-dried duck meat. The hydrocarbons had a higher threshold value, so their effects on the flavor formation of air-dried duck meat were not significant.

Taken together, the results revealed that PEF treatment significantly (*p* < 0.05) increased the diversity and concentration of key volatile compounds (especially aldehydes, esters, and alcohols) in air-dried duck meat, with the most pronounced effect at the shorter treatment time (30 s). It was confirmed that aldehydes, esters, and alcohols have very low flavor thresholds and important flavor properties that contribute significantly to the formation of the overall aroma of meat products [36]. In summary, the MPEF-30 and HPEF-30 treatment groups detected a higher content of flavor substances and a richer variety of flavor compounds compared to the other treatment groups.

To further analyze the contribution of different aroma components to differentiate air-dried duck meat in different treatment groups, four differential aroma substances including hexanal, methyl caprate, and 4-methyl valerate were screened according to the criteria of VIP > 1 and *p* < 0.05 (Figure 4C). The primary source of hexanal (grass, tallow, and fat flavors) [35] is lipid oxidation, particularly the breakdown of linoleic and linolenic acids that are specially formed during air-drying when exposed to air, with a very low threshold (4.5 μg/kg), which contributes to the freshness and fat flavor of the air-dried duck meat. Different studies have found that hexanal is the main aldehyde in cured meat products. Appropriate levels of hexanal do not impair the freshness of meat products and contribute some good flavor, but storage changes still need to be monitored. Methyl caprate (sweet, fruity flavors) is produced by the esterification of fatty acids with methanol, and its formation is promoted by microbial fermentation during the processing of air-dried duck meat, which enhances the mellow flavor and complexity of air-dried duck meat. 4-methyl valerate (fruity, cheesecake flavors) is a metabolite of branched-chain amino acid (leucine), which is closely related to microbial fermentation during processing and has a very low threshold value (about 1 μg/kg), which can enhance the flavor of air-dried duck meat.

### 3.8. FAA Analysis

The content of FAAs significantly affects the formation of the texture and taste quality of meat products. Combining the results of PEF treatment on the textural characteristics, moisture distribution, microstructure, endogenous protease activity, and GC-MS of air-dried duck meat, it was determined that PEF treatment with a low treatment time (30 s) not only improved the tenderness and water retention of air-dried duck meat and maintained a more intact tissue morphology, but also increased the activity of endogenous enzymes, promoted protein degradation, and enhanced the taste quality of air-dried duck meat. Therefore, the PEF-treated groups (LPEF-30, MPEF-30, and HPEF-30) with the better overall quality of air-dried duck meat were selected to determine their FAA content and to examine how PEF treatment affects the composition of FAAs and the taste activity value (TAV) of air-dried duck meat.

The content of FAAs in air-dried duck meat after PEF treatment is shown in Table 3. In contrast to the CK group, the total FAA content in the PEF-treated group increased significantly (*p* < 0.05), and the total FAA content in the air-dried duck meat of the MPEF-30 group reached 749.68 mg/100 g, which was 15.66% higher than that of the CK group. This is due to the electroporation effect of PEF treatment that destroys protein structure and promotes protein degradation, which leads to an increase in total free amino acid content or individual amino acid content (Liu et al., 2018 [37]). Kantono et al. (2021) [38] found that PEF treatment induced protein hydrolysis, which in turn increased free amino acid content (especially Ala, Phe, Gly, and Asp) in both chilled and freeze-thawed lamb. The study of Liu et al. (2024) [39] showed that PEF treatment considerably raised the amino acid content of air-dried New Zealand apricots (*p* < 0.05), and nearly all amino acid content rose as the strength of the electric field increased.

The contents of aspartic acid (Asp) and glutamic acid (Glu) were significantly increased in the PEF-treated group (*p* < 0.05), and Asp and Glu, as umami amino acids, can enhance the sensory characteristics (e.g., tenderness and juiciness) of meat and positively affect the meat quality [17]. The umami amino acid contents of the LPEF-30, MPEF-30, and HPEF-30 groups were 9.51%, 17.42%, and 6.06% greater than those of the CK group, respectively. Glycine (Gly), threonine (Thr), and alanine (Ala), as amino acids with sweetness perception, could confer pleasant taste, and the sweet amino acid content of the PEF-treated group accounted for 27–32%, which was 21.92%, 17.15%, and 12.88% higher than that of the CK group, respectively. It is noteworthy that the content of bitter amino acids (e.g., arginine, methionine) also showed an increasing trend in the PEF-treated group, which related to the protein denaturation and secondary bond breaking induced by the electric field action, resulting in the exposure of more bitter amino acid residues. Among them, leucine, although presenting bitter properties, is also an important precursor of taste compounds, being converted by transaminases to α-keto acids, which are subsequently metabolized into taste compounds [40,41]. It has been shown that the interaction of FAAs of different compositions and concentrations leads to differentiated taste profiles in the final product. Therefore, PEF treatment with appropriate electric field strength can significantly increase the fresh and sweet amino acids in air-dried duck meat, improve the taste characteristics of air-dried duck meat, and also promote the formation of good taste compounds.

The contributing roles of the taste substances were not only related to their contents but also depended on their taste activity thresholds. As shown in Table 3, only Glu and Ala had TAV values above 1, indicating that these two FAAs contributed directly to the taste of air-dried duck meat, and the TAV value of Glu was as high as 3.55, highlighting its central position in the formation of fresh taste substances. The TAV values of Val, His, and Lys were close to 1, indicating that these amino acids also had an important influence on the construction of the taste profile. In conclusion, PEF treatment significantly enhanced the freshness intensity of air-dried duck meat and effectively improved the taste profile of air-dried duck meat. In addition, most of the sweet amino acids and bitter amino acids were below their taste thresholds, which did not directly promote the flavor of the air-dried duck meat but could enhance the umami and sweetness of the air-dried duck meat through synergistic effects with other taste components, thus improving the overall taste quality of the air-dried duck meat.

## 4. Conclusions

In this research, the effects of PEF treatment on the textural characteristics and flavor formation of air-dried duck meat were examined by determining the color, textural characteristics, moisture distribution, microstructure, endogenous protease activity, GC-MS, and free amino acid content. The results showed that PEF treatment improved the brightness and textural properties of air-dried duck meat, where the HPEF-30 treatment group significantly reduced hardness (65.44%), chewiness (59.97%), and gumminess (60.57%). In addition, PEF treatment increased the moisture content of air-dried duck meat by 5.55–9.50%. The results of LF-NMR and SEM confirmed that the MPEF group promoted the cleavage and vacuolization of the muscle fibers of air-dried duck meat, which significantly increased the proportion of water that could not easily flow in air-dried duck meat, decreased the water freeness, and improved the water retention of air-dried duck meat. According to the results of endogenous protease activities, PEF treatment significantly boosted the activities of cathepsin-B and cathepsin-L, with increases of 163.71%, and 10.04% in the MPEF-30 treated group, respectively. The increase in endogenous protease activity promoted protein degradation and facilitated the production of flavor substances. GC-MS analysis revealed that the contents of aldehydes, esters, alcohols, and ketones in air-dried duck meat in the MPEF-30 group were elevated, which improved the flavor quality of air-dried duck meat. The free amino acid results showed that MPEF-30 treatment increased the total free amino acid content (15.66%), especially the production of fresh (Glu, Asp) and sweet amino acids (Gly, Thr, and Ala). The results of these studies provide theoretical support for the innovative application of PEF technology in air-dried meat products. In the future, the effects of PEF treatment in combination with other technologies on the edible quality of air-dried meat products can also be explored to promote industrial transformation and upgrading.

## Figures and Tables

**Table 1:** The effect of PEF treatment on the color of air-dried duck meat.

Code	Electric Field Strength (kV/cm)	Treatment Time (s)	L*	a*	b*
CK	-	-	37.98 ± 0.91 ^e^	10.72 ± 0.54 ^b^	11.41 ± 2.10 ^ac^
LPEF-30	1	30	44.75 ± 0.91 ^a^	11.11 ± 1.22 ^b^	10.74 ± 3.45 ^ac^
LPEF-60	1	60	40.45 ± 2.47 ^bd^	12.35 ± 1.79 ^ab^	12.03 ± 2.52 ^ab^
LPEF-90	1	90	38.54 ± 2.07 ^de^	11.90 ± 2.37 ^ab^	12.88 ± 2.49 ^a^
MPEF-30	2	30	41.92 ± 2.13 ^b^	11.17 ± 1.15 ^b^	10.20 ± 0.81 ^ac^
MPEF-60	2	60	38.27 ± 1.43 ^de^	11.76 ± 2.14 ^ab^	13.37 ± 1.73 ^a^
MPEF-90	2	90	38.89 ± 0.92 ^ce^	11.17 ± 1.31 ^b^	11.50 ± 1.57 ^ab^
HPEF-30	3	30	40.84 ± 1.40 ^bc^	11.70 ± 1.13 ^ab^	13.10 ± 1.60 ^a^
HPEF-60	3	60	40.27 ± 1.53 ^bd^	13.74 ± 1.19 ^a^	11.54 ± 1.52 ^ab^
HPEF-90	3	90	38.95 ± 0.94 ^ce^	10.79 ± 0.85 ^b^	11.88 ± 0.78 ^a^

Data are shown as mean ± S.E. Different letters (a–e) in the same column indicate significant differences in one-way ANOVA and Duncan’s multiple comparisons (*p* < 0.05). CK: untreated; LPEF-30: PEF treatment (1 kV/cm, 30 s); LPEF-60: PEF treatment (1 kV/cm, 60 s); LPEF-90: PEF treatment (1 kV/cm, 90 s); MPEF-30: PEF treatment (2 kV/cm, 30 s); MPEF-60: PEF treatment (2 kV/cm, 60 s); MPEF-90: PEF treatment (2 kV/cm, 90 s); HPEF-30: PEF treatment (3 kV/cm, 30 s); HPEF-60: PEF treatment (3 kV/cm, 60 s); and HPEF-90: PEF treatment (3 kV/cm, 90 s).

**Table 2:** The effects of PEF treatment on the texture characteristics and moisture content of air-dried duck meat.

Treatment	Hardness (g)	Chewiness	Gumminess	Moisture Content (%)
CK	1084.26 ± 235.18 ^a^	696.72 ± 176.19 ^a^	761.37 ± 191.23 ^a^	60.02 ± 1.78 ^b^
LPEF-30	792.98 ± 93.71 ^bc^	573.68 ± 112.84 ^ac^	603.35 ± 118.97 ^ab^	65.23 ± 1.16 ^a^
LPEF-60	800.00 ± 56.04 ^bc^	503.40 ± 68.54 ^ad^	552.15 ± 81.14 ^ab^	65.13 ± 0.52 ^a^
LPEF-90	898.95 ± 85.37 ^ab^	635.11 ± 93.40 ^ab^	668.23 ± 93.51 ^ab^	65.15 ± 1.21 ^a^
MPEF-30	724.78 ± 53.35 ^bc^	465.62 ± 52.53 ^bd^	505.78 ± 50.02 ^bc^	65.47 ± 1.93 ^a^
MPEF-60	709.37 ± 229.19 ^bc^	476.14 ± 131.40 ^bd^	503.35 ± 139.13 ^bc^	65.72 ± 0.18 ^a^
MPEF-90	721.41 ± 243.12 ^bc^	459.92 ± 173.16 ^bd^	494.25 ± 183.02 ^bc^	63.40 ± 2.04 ^a^
HPEF-30	374.74 ± 70.36 ^d^	278.89 ± 28.87 ^d^	300.21 ± 34.80 ^c^	65.40 ± 0.84 ^a^
HPEF-60	641.07 ± 203.23 ^bd^	443.47 ± 133.85 ^bd^	485.05 ± 158.29 ^bc^	63.35 ± 1.17 ^a^
HPEF-90	561.29 ± 117.82 ^cd^	404.41 ± 105.44 ^cd^	437.63 ± 110.99 ^bc^	63.97 ± 1.01 ^a^

Data are shown as mean ± S.E. Different letters (a–d) in the same column indicate significant differences in one-way ANOVA and Duncan’s multiple comparisons (*p* < 0.05).

**Table 3:** The effects of PEF treatment on the free amino acid content and TAV value of air-dried duck meat.

Free Amino Acid	Taste Attribute	Taste Threshold (mg/100 mL)	Free Amino Acid Content (mg/100 g)				TVA			
			CK	LPEF-30	MPEF-30	HPEF-30	CK	LPEF-30	MPEF-30	HPEF-30
Asp	Umami (+)	100	28.72 ± 0.80 ^b^	33.45 ± 0.77 ^a^	34.39 ± 0.75 ^a^	28.77 ± 0.40 ^b^	0.29	0.33	0.34	0.29
Glu	Umami (+)	30	91.32 ± 0.95 ^c^	98.00 ± 1.21 ^b^	106.56 ± 4.17 ^a^	98.55 ± 1.58 ^b^	3.04	3.27	3.55	3.29
Ser	Sweet (+)	150	10.21 ± 0.65 ^a^	4.92 ± 0.47 ^b^	10.97 ± 0.17 ^a^	10.20 ± 0.90 ^a^	0.07	0.03	0.07	0.07
His	Bitter (−)	20	18.17 ± 0.94 ^b^	18.50 ± 0.33 ^b^	19.87 ± 0.19 ^a^	17.06 ± 0.82 ^b^	0.91	0.93	0.99	0.85
Gly	Sweet (+)	130	35.58 ± 0.99 ^c^	36.15 ± 0.22 ^bc^	42.52 ± 1.25 ^a^	37.85 ± 0.94 ^b^	0.27	0.28	0.33	0.29
Thr	Sweet (+)	260	39.49 ± 1.54 ^b^	43.09 ± 0.57 ^ab^	44.92 ± 0.64 ^a^	41.06 ± 2.60 ^b^	0.15	0.17	0.17	0.16
Arg	Bitter (−)	50	33.85 ± 1.30 ^c^	34.13 ± 0.28 ^c^	39.75 ± 1.61 ^b^	44.54 ± 0.80 ^a^	0.68	0.68	0.80	0.89
Ala	Sweet (+)	60	92.18 ± 3.49 ^c^	132.20 ± 1.04 ^a^	109.47 ± 2.23 ^b^	111.18 ± 1.39 ^b^	1.54	2.20	1.82	1.85
Tyr	Bitter (−)	-	19.38 ± 1.06 ^c^	18.57 ± 0.28 ^c^	30.49 ± 0.29 ^a^	25.98 ± 0.52 ^b^	-	-	-	-
Cys	-	-	1.69 ± 0.09 ^a^	1.25 ± 0.04 ^b^	1.78 ± 0.05 ^a^	1.35 ± 0.02 ^b^	-	-	-	-
Val	Sweet/Bitter (+)	40	38.41 ± 1.24 ^b^	36.89 ± 0.13 ^bc^	35.47 ± 1.66 ^c^	41.65 ± 1.10 ^a^	0.96	0.92	0.89	1.04
Met	Bitter (−)	30	18.80 ± 0.79 ^b^	19.69 ± 0.12 ^b^	22.80 ± 0.65 ^a^	18.53 ± 1.41 ^b^	0.63	0.66	0.76	0.62
Phe	Bitter (−)	90	33.28 ± 1.40 ^b^	30.40 ± 0.36 ^c^	38.40 ± 0.32 ^a^	29.55 ± 1.22 ^c^	0.37	0.34	0.43	0.33
Ile	Bitter (−)	90	24.94 ± 1.17 ^b^	23.29 ± 0.13 ^c^	28.17 ± 0.57 ^a^	22.88 ± 0.73 ^c^	0.28	0.26	0.31	0.25
Leu	Bitter (−)	190	53.82 ± 1.67 ^b^	50.12 ± 0.33 ^c^	64.39 ± 1.21 ^a^	49.14 ± 1.64 ^c^	0.28	0.26	0.34	0.26
Lys	Bitter/Sweet (−)	50	42.03 ± 1.54 ^c^	36.30 ± 0.68 ^d^	49.98 ± 0.97 ^a^	45.86 ± 1.77 ^b^	0.84	0.73	0.99	0.92
Pro	Sweet/Bitter (+)	300	66.32 ± 1.09 ^a^	47.28 ± 1.20 ^c^	58.80 ± 1.99 ^b^	44.97 ± 0.06 ^d^	0.22	0.16	0.20	0.15
Total	-	-	648.19 ± 5.58 ^c^	664.23 ± 1.09 ^b^	738.71 ± 14.53 ^a^	669.12 ± 9.17 ^b^	-	-	-	-
Umami taste FAAs	-	-	120.04 ± 1.71 ^c^	131.45 ± 1.04 ^b^	140.95 ± 4.89 ^a^	127.31 ± 1.51 ^b^	-	-	-	-
Sweet taste FAAs	-	-	177.45 ± 3.83 ^d^	216.35 ± 0.94 ^b^	207.88 ± 3.98 ^a^	200.30 ± 1.98 ^c^	-	-	-	-
Bitter taste FAAs	-	-	149.59 ± 6.15 ^b^	140.87 ± 0.68 ^c^	170.34 ± 2.18 ^a^	155.61 ± 4.49 ^b^	-	-	-	-

Data are shown as mean ± S.E. Different letters (a–d) in the same row indicate significant differences in one-way ANOVA and Duncan’s multiple comparisons (*p* < 0.05).

## Data Availability

The original contributions presented in this study are included in the article/Supplementary Material; further inquiries can be directed to the corresponding author.

## Supplementary Materials

The following supporting information can be downloaded at https://www.mdpi.com/article/10.3390/foods14111891/s1. Table S1: Volatile flavoring substances in air-dried duck meat were analyzed qualitatively and quantitatively (ng/g) by GC-MS.
