# Supplementary material for: Effect of Pulsed Electric Field Pretreatment on the Texture and Flavor of Air-Dried Duck Meat
Source: Foods. 2025 May 26;14(11):1891. doi: 10.3390/foods14111891 (PMC12154340; doi:10.3390/foods14111891)
Supplement: Supplementary file 1 [file foods-14-01891-s001.zip › foods-3649071-supplementary.pdf]

Supplementary Table S1. Volatile flavoring substances in air-dried duck meat were analyzed qualitatively and quantitatively (ng/g) by GC-MS. Data are expressed as mean ± standard deviation. Different letters (a-g) in each line indicate statistically significant differences ($P<0.05$). CK: untreated; LPEF-30: 1 kV/cm, 30 s; LPEF-60: 1 kV/cm, 60 s; LPEF-90: 1 kV/cm, 90 s; MPEF-30: 2 kV/cm, 30 s; MPEF-60: 2 kV/cm, 60 s; MPEF-90: 2 kV/cm, 90 s; HPEF-30: 3 kV/cm, 30 s; HPEF-60: 3 kV/cm, 60 s; HPEF-90: 3 kV/cm, 90 s.

Volatile flavor compound	CK	LPEF-30	LPEF-60	LPEF-90	MPEF-30	MPEF-60	MPEF-90	HPEF-30	HPEF-60	HPEF-90
**Aldehydes**										
Hexanal	151.51 ± 16.64^{bc}	86.35 ± 38.56^{de}	47.57 ± 6.58^{ef}	33.16 ± 8.30^{f}	200.33 ± 7.76^{a}	115.38 ± 12.48^{cd}	134.74 ± 34.38^{bc}	196.29 ± 41.18^{a}	163.93 ± 16.59^{ab}	145.00 ± 30.59^{bc}
Heptanal	10.03 ± 2.19^{bc}	7.51 ± 3.73^{c}	2.00 ± 0.70^{d}	1.93 ± 0.64^{d}	12.80 ± 1.53^{b}	8.32 ± 1.89^{c}	9.44 ± 0.59^{bc}	17.10 ± 2.95^{a}	9.98 ± 1.98^{bc}	9.87 ± 2.63^{bc}
Octanal	19.16 ± 2.30^{bc}	15.71 ± 3.18^{c}	7.64 ± 2.81^{d}	6.80 ± 1.77^{d}	27.62 ± 6.31^{a}	17.59 ± 1.70^{bc}	17.69 ± 1.41^{bc}	22.99 ± 1.73^{ab}	17.79 ± 3.37^{bc}	19.92 ± 5.56^{bc}
Decanal	5.05 ± 0.59^{b}	3.71 ± 0.59^{cd}	2.91 ± 0.92^{d}	1.60 ± 0.22^{e}	6.93 ± 0.23^{a}	4.12 ± 0.33^{c}	2.85 ± 0.58^{d}	5.53 ± 0.43^{b}	3.55 ± 0.62^{cd}	1.80 ± 0.41^{e}
Nonanal	42.71 ± 7.28^{ac}	30.98 ± 10.48^{cd}	19.66 ± 6.28^{de}	16.11 ± 1.59^{e}	55.52 ± 10.84^{a}	38.10 ± 2.86^{bc}	48.47 ± 8.47^{ab}	50.98 ± 1.93^{ab}	37.98 ± 4.52^{bc}	51.53 ± 14.75^{ab}
**Alcohols**										
Cycloheptanol	4.19 ± 0.43^{a}	1.46 ± 0.56^{c}	-	-	4.22 ± 0.80^{a}	2.44 ± 0.80^{b}	2.80 ± 0.36^{b}	3.86 ± 0.75^{a}	2.09 ± 0.17^{bc}	2.93 ± 0.76^{b}
1-Octen-3-ol	n.d.	18.05 ± 3.06^{b}	-	-	36.32 ± 5.96^{a}	7.56 ± 1.04^{c}	-	33.05 ± 6.64^{a}	-	-
4-Methyl-1-pentanol	1.72 ± 0.53^{bc}	0.64 ± 0.10^{c}	-	-	3.22 ± 0.65^{b}	-	-	5.74 ± 2.77^{a}	-	-
Methylazoxymethanol acetate	-	0.57 ± 0.16^{a}	-	0.64 ± 0.10^{a}	0.50 ± 0.21^{a}	-	-	-	-	-
2-Methyl-1-pentanol	-	2.27 ± 0.36^{c}	0.75 ± 0.10^{d}	0.65 ± 0.30^{d}	6.02 ± 0.58^{a}	2.54 ± 0.46^{c}	2.84 ± 0.50^{c}	4.96 ± 0.58^{b}	2.80 ± 0.62^{c}	2.85 ± 0.99^{c}
Decanol	-	6.47 ± 2.98^{c}	-	0.36 ± 0.04^{d}	10.50 ± 0.28^{b}	-	-	13.87 ± 1.83^{a}	-	-
3,3,6-Trimethyl-4,5-heptadien-2-ol	-	0.86 ± 0.21^{b}	0.66 ± 0.22^{b}	-	3.51 ± 0.55^{a}	-	-	3.57 ± 0.30^{a}	-	-
**Esters**										
Methyl hexanoate	8.93 ± 0.91^{c}	3.63 ± 0.13^{g}	9.03 ± 0.69^{c}	4.93 ± 0.66^{f}	15.07 ± 0.17^{a}	12.33 ± 0.36^{b}	7.99 ± 0.07^{d}		7.02 ± 0.82^{e}	7.80 ± 0.05^{de}
Methyl heptanoate	-	-	0.36 ± 0.07^{a}	-	0.38 ± 0.20^{a}	-	-	-	-	-
Methyl octanoate	3.82 ± 0.66^{cd}	2.43 ± 0.16^{d}	3.87 ± 1.03^{cd}	1.68 ± 0.17^{d}	14.24 ± 3.09^{a}	5.14 ± 0.27^{c}	2.27 ± 0.09^{d}	9.03 ± 1.56^{b}	2.77 ± 0.25^{d}	3.56 ± 0.43^{cd}
Methyl nonanoate	-	-	0.47 ± 0.08^{b}	0.41 ± 0.08^{b}	1.76 ± 0.11^{a}	-	-	-	-	-
Ethyl 4-methyl valerate	8.61 ± 1.14^{c}	13.13 ± 0.44^{b}	3.13 ± 0.68^{d}		12.95 ± 1.44^{b}	3.47 ± 0.82^{d}		17.77 ± 1.23^{a}	4.63 ± 2.23^{d}	
Methyl caprate	11.87 ± 0.73^{ab}	9.69 ± 0.84^{b}	6.14 ± 1.53^{c}	3.55 ± 2.07^{cd}	13.01 ± 1.45^{a}	6.48 ± 2.98^{c}	2.66 ± 0.42^{d}	14.40 ± 2.52^{a}	9.69 ± 1.74^{b}	6.23 ± 1.91^{c}
Methyl tri-decanoate	-	0.80 ± 0.15^{c}	0.71 ± 0.06^{c}	0.40 ± 0.03^{c}	15.48 ± 3.55^{a}	-	-	9.69 ± 4.25^{b}	-	-
**Ketones**										

2-Heptanone	0.97±0.25^{d}	1.07±0.21^{cd}	0.51±0.17^{e}	0.42±0.18^{e}	2.01±0.26^{a}	1.31±0.09^{bd}	1.29±0.19^{bd}	1.76±0.26^{ab}	1.48±0.50^{bc}	1.41±0.30^{bd}
5-Methyl-2-hexanone	0.64±0.10^{cd}	0.86±0.16^{bc}	0.50±0.21^{cd}		1.16±0.05^{b}	0.59±0.08^{cd}		1.61±0.68^{a}	0.32±0.10^{de}	
N,N'-Bis(2-methyl-2-nitrosopentan-4-one)	1.49±0.33^{d}	9.03±2.63^{c}	5.65±1.24^{cd}	5.50±0.65^{cd}	27.72±10.29^{a}	21.73±3.13^{ab}	22.19±1.97^{ab}	18.95±1.31^{b}	-	27.01±2.78^{a}
N,N'-Bis(2-methyl-2-nitrosobutan-3-one)	-	-	0.11±0.05^{c}	-	38.53±8.49^{a}	-	-	18.95±1.31^{b}	-	-
**Hydrocarbons**										
Dimethylketene	-	-	-	2.24±0.52^{a}	-	-	0.86±0.16^{b}	-	-	-
7-Methyl-3,4-octadiene	-	-	-	-	-	-	1.16±0.05^{b}	-	-	2.03±0.21^{a}
2,5-Dimethyldecane	-	-	0.23±0.06^{a}	0.19±0.05^{ab}	-	0.23±0.10^{a}	0.18±0.10^{ab}	-	0.13±0.01^{b}	0.13±0.01^{b}
2-Methyldodecane	-	-	0.66±0.20^{a}	-	-	-	-	-	-	-
2,6,11-Trimethyldodecane	-	-	0.58±0.13^{a}	0.34±0.00^{b}	-	-	-	-	-	-
13-Oxabicyclotridecane	-	-	-	2.18±0.86^{a}	-	-	1.27±0.03^{b}	-	-	1.56±0.42^{b}
2-Methyltridecane	3.59±0.19^{bc}	3.27±0.33^{c}	-	-	4.41±0.97^{ab}	3.44±0.15^{bc}	4.06±0.50^{ac}	4.73±0.63^{a}	3.38±0.55^{c}	4.01±0.78^{ac}
3-Methylheptadecane	8.27±0.86^{bc}	9.08±1.26^{ac}	5.95±2.60^{cd}	3.68±0.93^{d}	11.84±3.10^{ab}	7.63±2.98^{c}	6.29±1.31^{cd}	12.59±2.71^{a}	6.69±1.37^{cd}	5.93±1.23^{cd}
